# Large-scale prediction of adverse drug reactions-related proteins with network embedding

**DOI:** 10.1093/bioinformatics/btac843

**Published:** 2022-12-29

**Authors:** Jaesub Park, Sangyeon Lee, Kwansoo Kim, Jaegyun Jung, Doheon Lee

**Affiliations:** Department of Bio and Brain Engineering, Korea Advanced Institute of Science and Technology, Daejeon 34141, South Korea; Department of Bio and Brain Engineering, Korea Advanced Institute of Science and Technology, Daejeon 34141, South Korea; Department of Bio and Brain Engineering, Korea Advanced Institute of Science and Technology, Daejeon 34141, South Korea; Graduate School of Medical Science and Engineering, Korea Advanced Institute of Science and Technology, Daejeon 34141, South Korea; Department of Bio and Brain Engineering, Korea Advanced Institute of Science and Technology, Daejeon 34141, South Korea

## Abstract

**Motivation:**

Adverse drug reactions (ADRs) are a major issue in drug development and clinical pharmacology. As most ADRs are caused by unintended activity at off-targets of drugs, the identification of drug targets responsible for ADRs becomes a key process for resolving ADRs. Recently, with the increase in the number of ADR-related data sources, several computational methodologies have been proposed to analyze ADR–protein relations. However, the identification of ADR-related proteins on a large scale with high reliability remains an important challenge.

**Results:**

In this article, we suggest a computational approach, Large-scale ADR-related Proteins Identification with Network Embedding (LAPINE). LAPINE combines a novel concept called single-target compound with a network embedding technique to enable large-scale prediction of ADR-related proteins for any proteins in the protein–protein interaction network. Analysis of benchmark datasets confirms the need to expand the scope of potential ADR-related proteins to be analyzed, as well as LAPINE’s capability for high recovery of known ADR-related proteins. Moreover, LAPINE provides more reliable predictions for ADR-related proteins (Value-added positive predictive value = 0.12), compared to a previously proposed method (*P* < 0.001). Furthermore, two case studies show that most predictive proteins related to ADRs in LAPINE are supported by literature evidence. Overall, LAPINE can provide reliable insights into the relationship between ADRs and proteomes to understand the mechanism of ADRs leading to their prevention.

**Availability and implementation:**

The source code is available at GitHub (https://github.com/rupinas/LAPINE) and Figshare (https://figshare.com/articles/software/LAPINE/21750245) to facilitate its use.

**Supplementary information:**

Supplementary data are available at *Bioinformatics* online.

## 1 Introduction

Adverse Drug Reactions (ADRs) are defined as unintended harmful or unpleasant reactions caused by drug intervention (Coleman and Pontefract, 2016). Some serious ADRs are enough to result in life-threatening events or even death. ADRs impose a significant public health concern, contributing to over 100 000 deaths per year in the U.S (Ernst and Grizzle, 2001). Also, serious ADRs such as liver and kidney failures lead to drug withdrawals from the market (Qureshi *et al.*, 2011). Still, the prevention of ADRs is difficult due to an incomplete understanding of ADRs, which leads to an increase in the cost of drug development and the failure rates during clinical trials (Waring *et al.*, 2015). Therefore, complete and accurate information on drug ADRs is required to improve drug safety for patients and to reduce risks for pharmaceutical companies.

It is widely accepted that there are two types of ADRs; one refers to uncommon and idiosyncratic responses that cannot be predicted from known pharmacology, and the other to predictable responses caused by on-target or off-target interactions between drugs and proteins (Garon *et al.*, 2017; Pirmohamed *et al.*, 1998). Therefore, most previous studies on the subject of ADRs aim to mitigate the latter. From the early stages of drug discovery, pharmaceuticals have screened drug candidates binding against a panel of safety targets to anticipate possible ADRs (Bowes *et al.*, 2012; Whitebread *et al.*, 2005). However, these in vitro experimental tests for drug–target interactions are expensive, labor-intensive and time-consuming. Hence, in silico models such as molecular docking simulations or structure-activity relationship approaches have been proposed to calculate the binding affinity between drug and protein to predict the drug–target–ADR relationships (Wallach *et al.*, 2010; Yang *et al.*, 2009, 2011). Even then, these methods are available only to a few proteins with known three-dimensional structures.

To broaden the scope of the investigation to include more proteins, systematic methods were suggested to predict ADR-related drug targets. Some studies identified significantly correlated drug targets to ADR by combining drug–target interaction data and drug–ADR relation data (Kuhn *et al.*, 2013; Smit *et al.*, 2021). These methods successfully increased the number of proteins considered for relation with ADRs compared to previous structure-based methods. However, these methods were heavily dependent on the drug–target interaction data, such that proteins without known interaction with drugs were out of scope. Another study suggested an algorithm to infer potential ADR–protein relations based on an integrated network with protein–protein interactions, ADR–ADR similarities and ADR–protein relations (Chen *et al.*, 2016b). Although this large-scale prediction algorithm could consider all targetable proteins, there was still room for improvement in prediction accuracy due to the lack of information about the known ADR-related proteins which is the key of the algorithm. Furthermore, with the increase in the number of studies about ADRs, databases containing relations between proteins and potential ADRs were built by integrating information from ADR-related public databases and literature mining (Galletti *et al.*, 2021; Huang *et al.*, 2018). Despite their efforts, relations reported by these databases contain only a limited extent of ADRs and proteins.

To overcome the limitations of previous studies, we have focused on the recent advances in various techniques developed to embed biological information, especially network embedding techniques. Network embedding learns a low-dimensional representation for each node in the network without manual feature selection. By projecting every node in the given network with preserving the structural information, network embedding allows for the extraction of latent features of nodes. These features can be applied to many tasks related to networks, such as link prediction, community detection and node classification (Hamilton *et al.*, 2017). Also, network embedding techniques have been utilized for biomedical challenges such as predicting drug–drug interactions (Zhang *et al.*, 2018), protein–protein interactions (Wang *et al.*, 2017), drug–target interactions (Mohamed *et al.*, 2019), drug–disease associations (Zhou *et al.*, 2020) and disease similarity (Li *et al.*, 2021). So, through many previous studies, it has been shown that network embedding techniques can successfully extract node features from various biological networks.

**Fig. 1.**
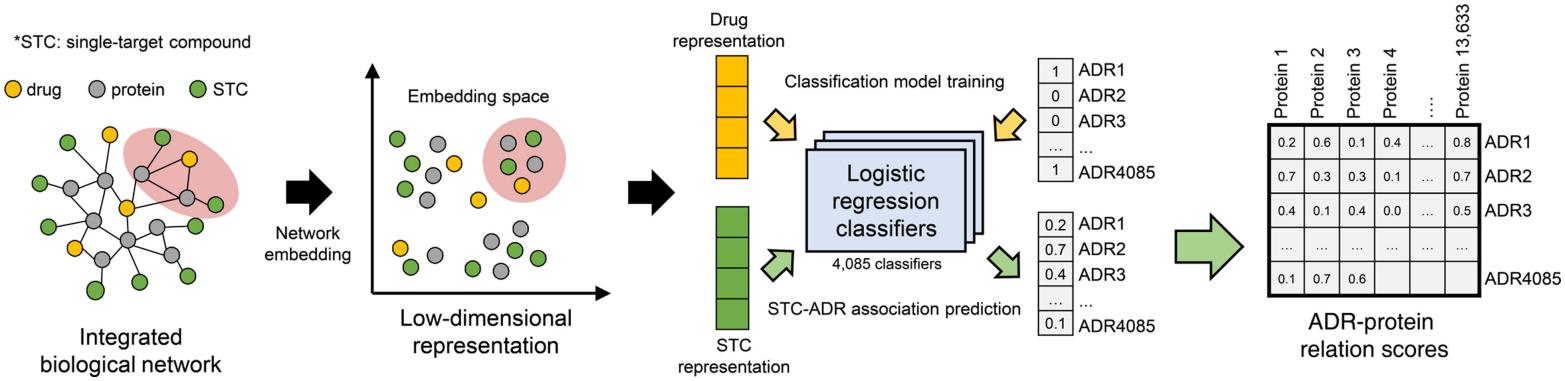
Overall process of LAPINE to predict ADR-related proteins

In this article, we suggest a computational approach, Large-scale ADR-related Proteins Identification with Network Embedding (LAPINE) that adopts the network embedding technique to develop a novel method to achieve large-scale prediction of ADR-related proteins (Fig. 1). One of the major challenges in predicting ADR-related proteins is that the number of known protein-ADR relations (2055 pairs in our benchmark dataset), available to train a predictive model, is very small compared to all possible relations to be predicted. The number of known ADR-related drugs, on the other hand, is relatively larger [139 756 pairs in the SIDER Database (Kuhn *et al.*, 2016)] and hence the accuracy of the model predicting drug–ADR relations is expected to be higher. Since each drug functions through its target proteins, we can thus try to transfer the relatively richer information from drug–ADR relations to protein-ADR relations, in order to overcome the limitations of the previous methods. However, it would not make sense to simply use embedding for proteins as the input for the drug–ADR models. We, therefore, introduced a novel concept called single-target compounds (STCs). An STC is a fictitious compound that has only one specific target protein, and likewise, each target protein has a corresponding STC. Then, we can assume that the biological effects of a given STC are entirely derived by perturbation started from its respective target protein. Therefore, if we can predict the likelihood that an STC causes ADRs, we can interpret it as the significance of the relation between the target protein of the STC and ADRs.

To this end, we constructed the integrated network containing drug–target interactions, protein–protein interactions and STC-protein interactions. Through the network embedding, we obtained low-dimensional representations of nodes for drugs and STCs in the integrated network. Then we trained logistic regression classifiers to predict the ADRs of the drugs by using low-dimensional representations of drugs as an input feature vector. Finally, the trained classifier was used to calculate the ADR probability of STCs, which can be interpreted as the score of the relation between ADR and a target protein of an STC. With LAPINE, we achieved ADR-related protein prediction on a larger scale than with previous approaches (Supplementary Table S5). Also, we illustrated that previously reported ADR–protein relations resulted in high scores and that drugs that bind to high-scoring ADR-related proteins exhibited ADRs more frequently compared to a previous study. Furthermore, additional literature investigation confirmed the reliability of the predicted ADR–protein relations.

## 2 Materials and methods

### 2.1 Collection of known ADR–protein relations as benchmark datasets

We collected a benchmark dataset of ADR–protein relations from three different previous studies. Kuhn *et al.* (2013) extracted over 200 ADR-target protein relations from PubMed’s abstract through manual curation, and Smit *et al.* (2021) collected and organized relations from three other papers about the drug-safety target. ADReCS-Target (Huang *et al.*, 2018) is a public database that provides information about ADRs caused by interaction between drugs and proteins, manually curated from the MEDLINE database. ADR terms are mapped to Preferred Terms (PTs) suggested by Medical Dictionary for Regulatory Activities Terminology (MedDRA) (Brown *et al.*, 2012), and proteins are mapped to Ensembl protein ID. Finally, we collected 218 ADR–protein relations from (Kuhn *et al.*, 2013), 964 from (Smit *et al.*, 2021) and 905 from the ADReCS-Target.

### 2.2 Collection of protein–drug, protein–protein interactions and drug–ADR relations

We obtained protein–protein interaction data from STRING (Szklarczyk *et al.*, 2021), a database with an extensive collection of known and predicted physical or functional associations between proteins. Also, we obtained drug–protein interaction data from STITCH (Szklarczyk *et al.*, 2016), which integrated information about interactions between chemicals and proteins extracted from metabolic pathways, experimental results and other databases. For both STRING and STITCH databases, we only considered interactions that had been experimentally verified or extracted from other public databases. Also, as both databases assigned confidence scores (0–1000) to each interaction according to the evidence, we set a threshold value of 700 for the confidence score, as recommended by the authors as a ‘high confidence score’. This value was also demonstrated by achieving the highest Pearson correlation coefficient between ADR similarity and target similarity of drug pairs (Supplementary Fig. S1). As a result, we extracted 702 834 protein–protein interactions between 13 633 proteins and 32 964 drug–protein interactions between 1136 drugs and 6952 proteins.

We obtained drug–ADR relations from the SIDER database (Kuhn *et al.*, 2016) which contains information on marketed drugs and their ADRs extracted from public documents and medical prescribes. The ADR terms in SIDER are mapped to MedDRA Preferred Terms. Drugs that have no target proteins in the STITCH database were excluded from our analysis. Hence, ADRs not related to the rest of the drugs were also excluded from the analysis. To summarize, the LAPINE utilized 1103 drugs and 4085 ADRs.

### 2.3 Construction of the integrated network of drug, protein and STC

The integrated network was constructed by combining protein–protein interactions and drug–protein interactions. In particular, drug–protein interactions were parsed to include only those between drugs with at least one ADR and proteins that are present in the PPI network. The directions for the edges were determined to take into account the characteristics of each biological interaction. Hence, drug–protein interactions were converted into directed edges from drug to protein, while protein–protein interactions were converted into bidirectional edges in the integrated network. For all proteins in the network, one STC targeting the corresponding protein was added to the network. Interactions between STCs and proteins were also converted into directed edges from STC to protein. The final integrated network consists of 1103 drugs, 13 633 proteins and 13 633 STCs with 1 444 902 directed edges between them.

### 2.4 Network embedding of the integrated network

We used node2vec in embedding the integrated network to obtain low-dimensional representations of drugs and STCs in the same embedding space. The node2vec (Grover and Leskovec, 2016) is a flexible network embedding model which learns representations of nodes based on sequences of nodes sampled through random walks. Given the assumption that nearby nodes in the random walk sequence are neighbors, node2vec learns a low-dimensional vector representation of each node that maximizes the likelihood of preserving the node’s neighbors in the network. In the following ADR prediction process, we confirmed the suitability of node2vec for our study compared to four other embedding methods (Cao *et al.*, 2015; Kipf and Welling, 2016; Ou *et al.*, 2016; Tang *et al.*, 2015) which showed high performance in the node classification task reviewed by Yue *et al.* (2020) (Supplementary Fig. S2).

The integrated network used for embedding was considered directed and weightless according to the previously described properties. The hyperparameters *p* and *q* that determine the characteristics of the biased random walk in node2vec were optimized with values that maximize the performance of the subsequent ADRs prediction model (Supplementary Table S1). Other hyperparameters of node2vec were set to values as recommended by a previous study (Yue *et al.*, 2020).

### 2.5 Learning the ADR prediction model and prioritizing ADR-related proteins

Predicting multiple ADRs for a drug is generally considered a multi-label classification task. For our research, we transformed this into a set of independent binary classification tasks, each with logistic regression classifiers based on the binary relevance method (Tsoumakas *et al.*, 2006). Logistic regression classifiers were trained for each ADR with the low-dimensional representation of drugs from network embedding as the input. So we trained the identical number of classifiers for all 4085 ADRs. The drug–ADR relations extracted from SIDER were used as a training set for learning. After the training was completed, relations between each STC and ADRs were predicted by applying the model to the low-dimensional representation of the STC. The predicted STC-ADR relation score then can be interpreted as a relation score between the target protein of the STC and the ADR. Hence, ranks of the scores of all proteins for the ADR is used to prioritize the proteins related to the ADR.

To compare embedding algorithms and optimize hyperparameters for the classifier, we trained the ADR prediction model using 10-fold cross-validation. The SIDER dataset is split into 10 sets, in each fold one set is used as test data and the other set is used as training data. To enable 10-fold cross-validation, ADRs with less than 10 drugs were excluded. We measured the area under the ROC curve (AUROC) and the average precision (AP) for each ADR and averaged them to evaluate the performance of the model. The AP, which summarizes a precision-recall curve, was calculated as the weighted mean of precisions achieved at each threshold (Pedregosa *et al.*, 2011). In both metrics, higher values indicate better performance.

By comparing the distribution of performances, we selected node2vec as the best embedding method and set the regularization coefficient of the logistic regression to 0.1 (Supplementary Figs S2 and S3, Supplementary Table S1). All other parameters of logistic regression were set to default as recommended by the Scikit-Learn Python package (Pedregosa *et al.*, 2011). After these processes, we used all drug–ADR relations in the SIDER dataset to train the final model using optimized settings.

### 2.6 Comparison of ADR–protein relation prediction performance with other methods

We evaluated the prediction performance of LAPINE in comparison to those of previously suggested ADR–protein relation prediction models on the carefully constructed test dataset. Since the scope of each previous study is quite different, from the benchmark protein-ADR datasets, the positive samples were selected from ADRs that are common to LAPINE and previous methods. The negative samples however are not available from the such dataset, as we cannot state for certain that there is no relation between given pairs of ADRs and proteins. Thus, we considered non-positive samples among the relations between proteins and ADRs included in the benchmark dataset as negative samples for training models. Finally, we constructed a balanced test set by sampling randomly from the negative samples to the same number of positive samples. And the distribution of prediction performance for each test set was reported by repeating negative sampling 1000 times. Performance was calculated with AUROC and AP, as described in the previous section.

### 2.7 Quantitative evaluation of predicted proteins using drug–ADR relations and drug–target interactions

For each predicted protein, the significance of relations with each ADR can be inferred by calculating how many drugs that target the protein cause the ADR. In other words, for each ADR–protein pair, we can construct a confusion matrix that organizes the drugs into different categories, assuming the protein as a predictor of ADR and the ADR as the target event (Supplementary Fig. S4). With this matrix, we used two widely used indicators, positive predictive value (PPV) and likelihood ratio (LR) to evaluate the reliability of the predicted ADR–protein relations (Grimes and Schulz, 2005; Kuhn and Johnson, 2013).

PPV is the ratio of drugs showing ADR among drugs targeting the protein [PPV = TP/(TP + FP)]. Therefore, it can be considered that the ADR–protein relation with high PPV has high reliability (Smit *et al.*, 2021). However, the absolute value of PPV is severely affected by the overall prevalence of ADR. Therefore, to evaluate all ADRs with the same criteria, value-added PPV(VAPPV) (Coulthard, 2007), which is the value obtained by subtracting prevalence from PPV, was used as an evaluation index for the ADR–protein relation. LR is the calculation of how many times more likely an ADR-related drug binds to a target protein compared to other drugs [LR = TP×(FP + TN)/FP×(TP + FN)]. So, LR indicates how useful the target protein is in predicting the ADR of the drug (Grimes and Schulz, 2005). So, for quantitative evaluation of predictions from LAPINE, we calculated the VAPPV and LR of the top 50 predictive proteins according to their relation scores. But ADR–protein relations consisting of less than five drug-related proteins or less than five drug-related ADRs were excluded for statistical significance (Kuhn *et al.*, 2013).

### 2.8 Literature investigation and gene set enrichment analysis on the predicted proteins

In order to validate whether predicted proteins from LAPINE are indeed related to a given ADR, we searched for evidence by querying the related keywords in PubMed. This literature investigation process was done at two different depths. First, relations between an ADR and each of the top 10 predictive proteins were searched together to find the previously published research that indicated their relations. Each pair in which the relation can be attributed to direct causation was separately specified. Then, the same process was repeated at a different depth, with the top 10 KEGG pathways that were enriched by the top 50 predictive proteins. The enrichment test was performed via the Enrichr (Kuleshov *et al.*, 2016), and the top potential pathways were selected by *P*-value. The literature manual curation was done once again to look for publications with any relations between a given ADR and the process indicated by the KEGG pathways.

## 3 Results

### 3.1 Wide coverage of LAPINE verified by benchmark datasets

We obtained benchmark datasets from three different previous studies to evaluate our predictions. Among the previously reported ADR–protein relations, we calculated the percentage of relations in which the protein was not the target of any drugs related to the ADR (Table 1). Almost half of the proteins in benchmark datasets are not known targets of ADR-related drugs. Previous ADR-related protein identification methods that are heavily dependent on drug–target interaction data cannot analyze relations involving those proteins. However, as LAPINE can analyze all proteins in a protein–protein interaction network, it covers over 90% of all known ADR–protein relations in benchmark datasets. In conclusion, such statistics demonstrate the need for a new methodology that does not depend on drug–target interaction data for a complete understanding of proteins that trigger ADRs.

**Table 1.** Statistics of benchmark datasets of ADR–protein relations

Source			Drug target based methods (%)^a^	LAPINE (%)^b^
Kuhn’s dataset	No. of relations	218	126 (57)	150 (68)
Kuhn *et al.* (2013)	No. of ADRs	144	92 (63)	111 (77)
	No. of proteins	95	59 (62)	68 (71)
Smit’s dataset	No. of relations	964	509 (52)	883 (91)
Smit *et al.* (2021)	No. of ADRs	270	163 (60)	236 (87)
	No. of proteins	90	65 (72)	90 (100)
ADReCS-Target	No. of relations	905	275 (30)	679 (75)
Huang *et al.* (2018)	No. of ADRs	306	132 (43)	245 (80)
	No. of proteins	416	139 (33)	342 (82)

a Number of relations (ADRs, proteins) that can be analyzed in the prediction method based on drug target information.

b Number of relations (ADRs, proteins) that can be analyzed in the LAPINE.

### 3.2 Drugs with similar ADRs have similar low-dimensional representations through a network embedding

**Fig. 2.**
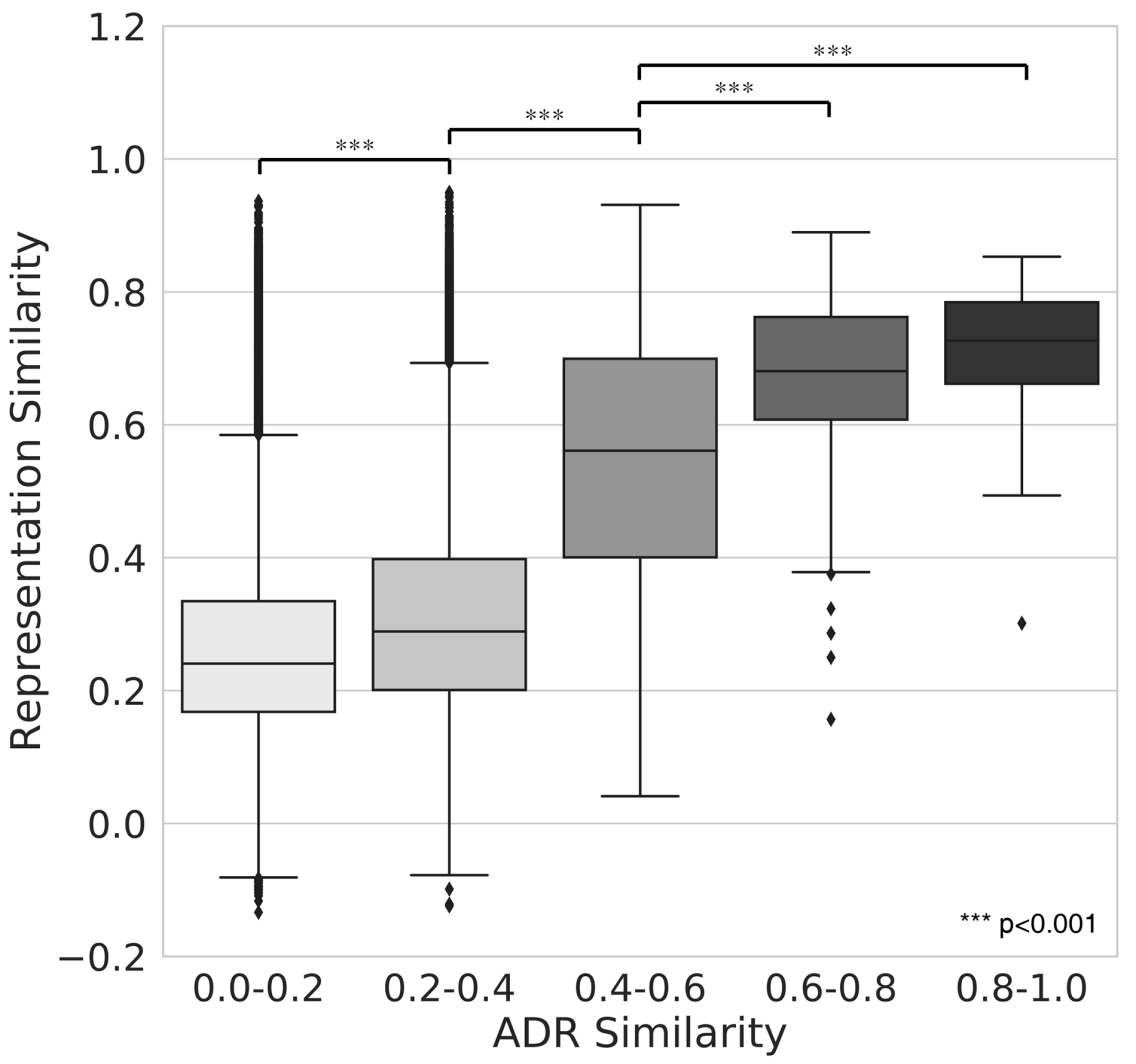
Correlation between ADR similarity and low-dimensional representation similarity of drug pairs. Box plots showing distributions of low-dimensional representation similarity of drug pairs for a specific range of ADR similarity

The integrated network, constructed by combining the protein–protein interactions, drug–protein interactions and STCs was embedded using the node2vec algorithm. As a result, we obtain low-dimensional representations of drugs and STCs in the integrated network. To evaluate how well the low-dimensional representations contain the pharmacological properties of drugs, we investigated the correlation between the similarity of the drug representations and the ADRs of drugs. Jaccard index was calculated for ADR similarity, and cosine similarity was calculated for drug representation similarity. The result shows that there is a correlation between the similarity of the ADRs of the two drugs and that of the drug vector representations (Fig. 2), implying that these low-dimensional vectors can be used to represent the pharmacological properties.

Meanwhile, among drug pairs with low ADR similarity, the high similarity of drug representations was observed in some cases. Considering the nature of the currently available ADR information (i.e. positive-unlabeled data), such cases suggest that the drug representations may imply unknown associations with ADRs. The pharmacological similarity of such drug pairs also supports this interpretation (Supplementary Table S2). In conclusion, the low-dimensional representation of drugs acquired by utilizing STCs in the integrated network can be utilized as a feature vector of drugs used for ADR prediction.

### 3.3 ADR prediction model with low-dimensional representations of drugs show reliable performance

**Fig. 3.**
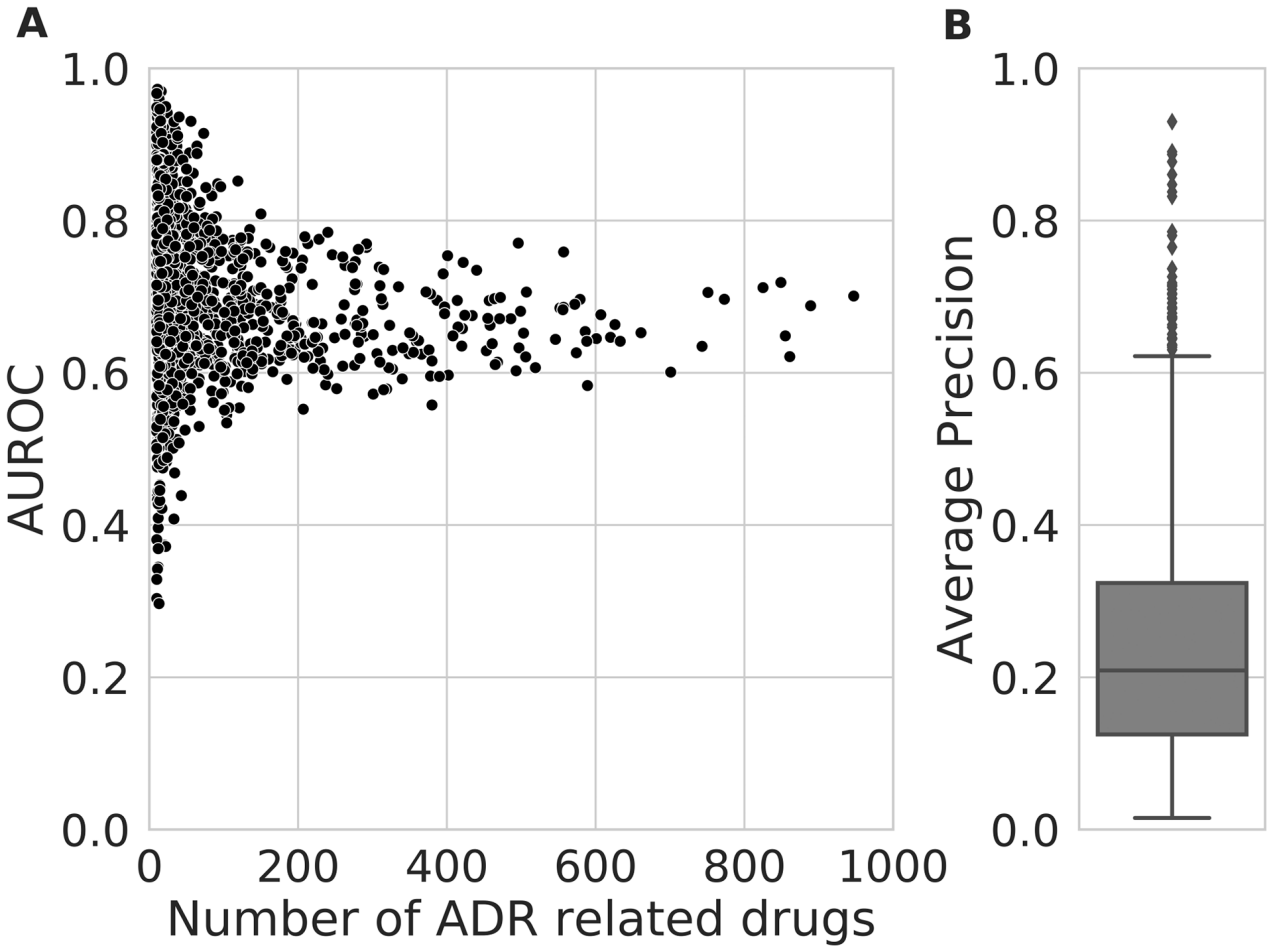
ADR prediction performance. (**A**) Scatterplot showing the distribution of AUROC values for each ADR according to the number of drugs involved in the ADR. (**B**) Boxplot showing the distribution of AP values for ADRs

By using the representation of the drug as an input feature vector, logistic regression classifiers were trained to predict the association between 1103 drugs and 4085 ADRs. To confirm the successful training of the ADR classification model, we evaluated the predictive performance for each ADR based on the 10-fold cross-validation. We used AUROC and AP as evaluation metrics, and each metric was calculated for each ADR and then averaged. Our ADR classification model achieves a macro-average AUROC of 0.70 and a macro-average AP of 0.24, exceeding the baseline performance (Fig. 3). In particular, the model showed more stable prediction performance when sufficient data on ADR-related drugs were provided. These results confirm that the proposed ADR prediction model was properly trained.

### 3.4 Previously reported ADR-related proteins are highly ranked in LAPINE

**Fig. 4.**
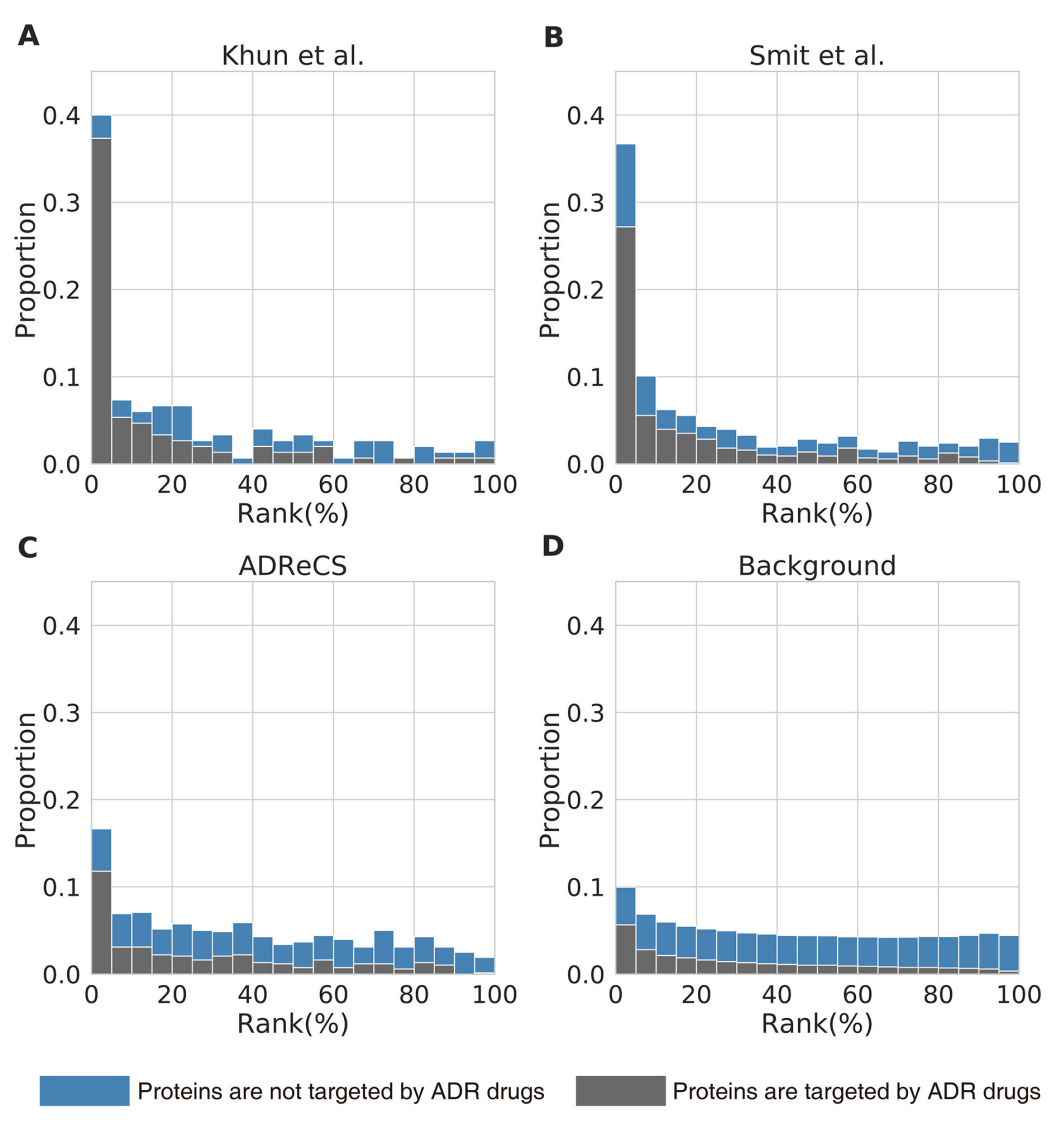
Retrieve known ADR–protein relations from benchmark datasets. (**A**, **B**, **C**) Histogram showing the rank distributions of ADR-related proteins predicted by LAPINE. Separate figures were created depending on the source of the dataset. In the histogram, one bar represents 5% of the total rank. (**D**) Histogram showing the rank distributions of ADR-related proteins of all proteins in benchmark datasets for all ADRs

Using the trained ADR prediction model and the low-dimensional representation of STC, the relations between the target protein of STC and ADRs were scored. To evaluate the reliability of the predicted ADR–protein relation scores from LAPINE, we examined the ranks for the previously reported ADR-related proteins in benchmark datasets. Protein ranks were calculated separately for each ADR and then converted to a percentage for the number of all proteins. The ADR-related proteins reported in 40% of Kuhn’s dataset, 30% of Smit’s dataset and 18% of the ADReCS-Target database ranked in the top 5% of all proteins, and more than half of all reported ADR-related proteins ranked in the top 20% (Fig. 4A–C). Also, 24% of all reported ADR-related proteins ranked in the top 5% were not targeted by drugs, which cannot be predicted with the methods dependent on drug–target interaction data.

In addition, we calculated the rank distribution of the scores of all proteins in the benchmark dataset for all predictable ADRs in LAPINE (Fig. 4D). The results indicated that the proteins in the benchmark dataset do not always have high relation scores for all ADRs. Hence, we can see that the high rank of reported ADR-related proteins is not the result of biased benchmark datasets which includes only a small number of proteins.

### 3.5 Comparison with other methods shows the improved performance of LAPINE

**Fig. 5.**
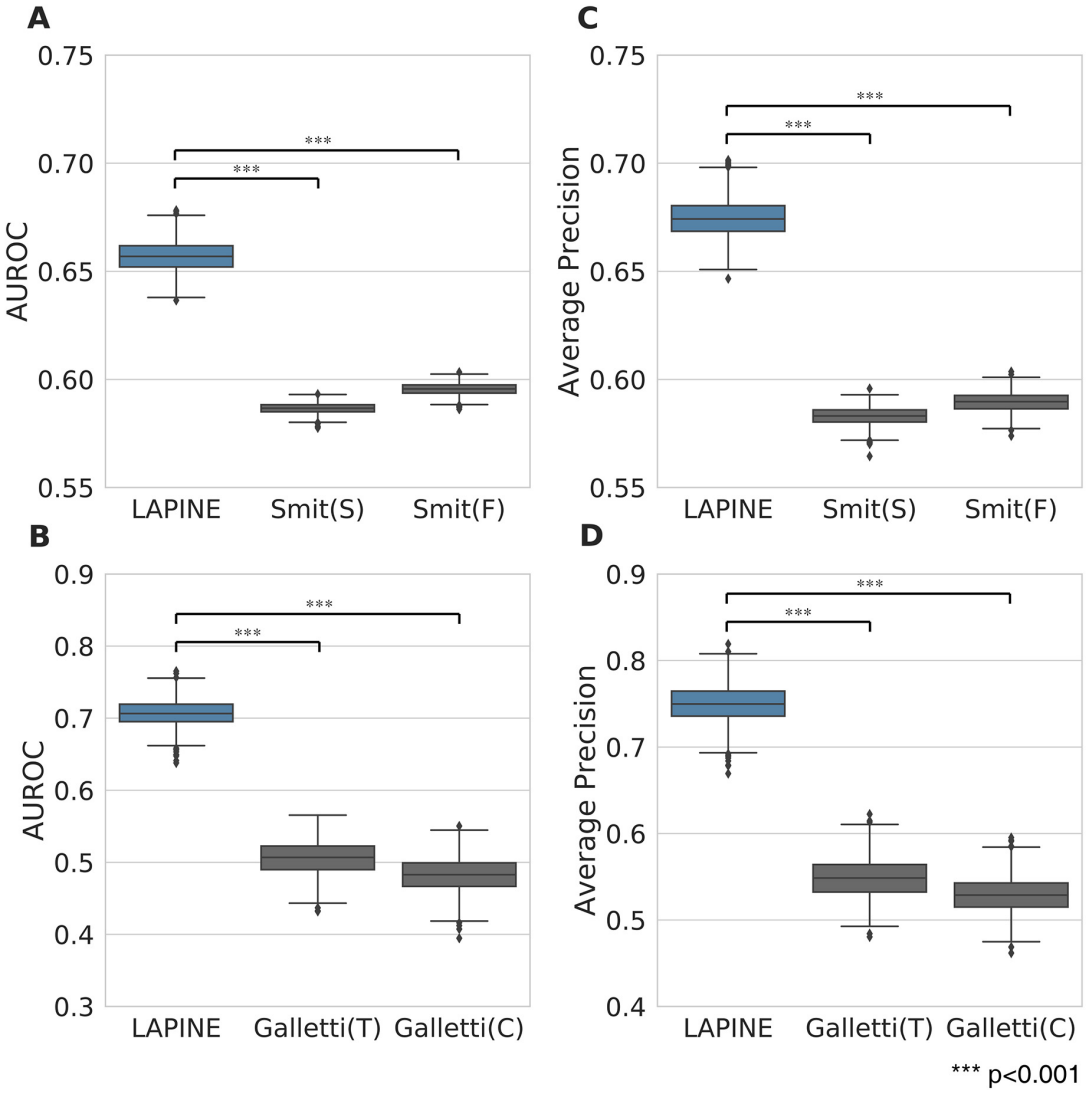
Evaluation of prediction performance of LAPINE by comparing with previously suggested ADR–protein relation prediction models. Box plots showing the distribution of AUROC and AP values across 1000 test sets constructed by random sampling. (**A**, **C**) (Smit *et al.*, 2021) reported two prediction results using two distinct databases: FAERs database(Smit(F)) and SIDER database(Smit(S)). The two box plots shown in the figure represent the performance for each result. (**B**, **D**) (Galletti *et al.*, 2022) reported two prediction results using two distinct datasets: The community dataset(Galletti(C)) and the controlled dataset(Galletti(T)). The two box plots shown in the figure represent the performance for each result

In this section, we compared the performance of LAPINE with two state-of-the-art methods (Galletti *et al.*, 2022; Smit *et al.*, 2021). The first method identified statistically significant ADR–protein relations based on ADR–drug–protein relations, while the second one predicted novel ADR–protein relations with an ensemble classification model learned with known ADR–protein relations. Since the scope of each previous study is quite different, we used different test sets for each comparison (see Section 2 for details). As a result, the test set for comparison with the method suggested by (Galletti *et al.*, 2022) contains 120 positive samples with 36 ADRs, and the test set for comparison with the method suggested by (Smit *et al.*, 2021) contains 1071 positive samples with 238 ADRs. As the result, there are two outcomes presented for each previous study, because both studies reported two distinct prediction outcomes depending on the dataset they used. From the results, we can see that LAPINE outperformed the two previous methods (Fig. 5). LAPINE achieved 0.657 and 0.675 of average AUROC and average AP respectively in one test set (Fig. 5A and C), and 0.706 and 0.749 for the other (Fig. 5B and D), which are significantly better than those for both previous methods (*t*-test, *P* < 0.001 for both). These results show that LAPINE overcame the important limitations of previous studies, and improved the performance in predicting the ADR–protein relations.

### 3.6 Drugs targeting high-ranked proteins were more likely to be associated with ADR

**Fig. 6.**
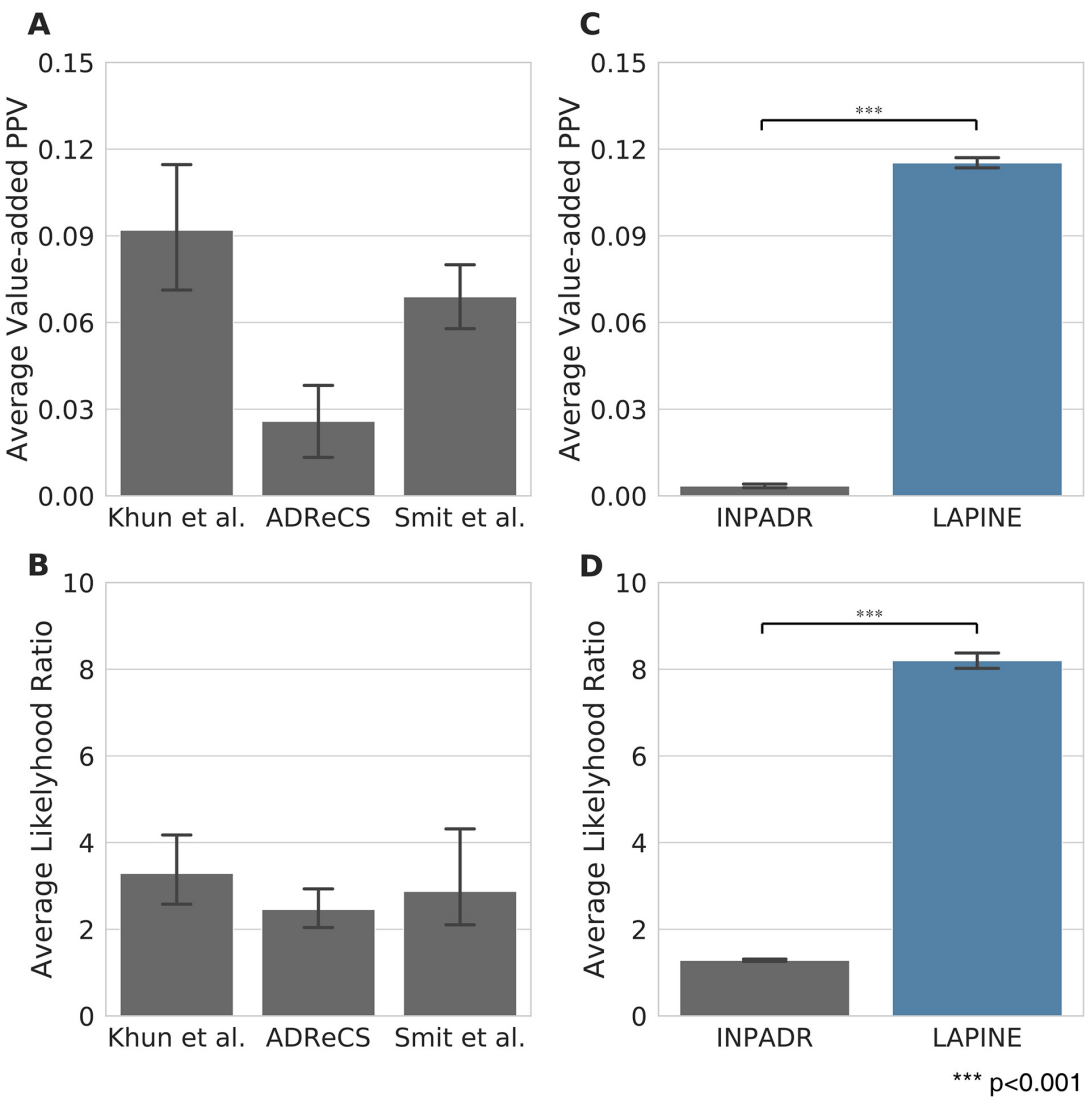
Evaluation of predicted ADR-related proteins using drug–target information. (**A**, **B**) Bar plots showing the average of VAPPV and LR of ADR–protein relations in each dataset. (**C**, **D**) Bar plots showing the average of VAPPV and LR of the top 50 predictive proteins for common ADRs. The error bar indicates the 95% confidence interval of the average

In order to quantitatively evaluate how reliable and useful the predicted ADR–protein relations are, we analyzed how often an ADR occurs in drugs targeting specific proteins. Obviously, drugs targeting ADR-related proteins should cause the related ADR more frequently than other drugs do. Such tendency can be measured with VAPPV and LR which are widely used metrics in clinical diagnosis (Grimes and Schulz, 2005; Kuhn and Johnson, 2013). To confirm the effectiveness of these metrics for evaluating the predicted ADR–protein relations, we calculated them on benchmark datasets and found that the average of VAPPV and LR were higher than the baseline (0 for VAPPV; 1 for LR) (Fig. 6A and B).

After confirming that both metrics characterize known ADR–protein relations, we calculated the representative VAPPV and LR by averaging the top 50 predictive proteins for each ADR. Then, we compared the prediction result with that from INPADR suggested by (Chen *et al.*, 2016b). To the best of our knowledge, INPADR is the only previously proposed large-scale ADR-related protein prediction method that can prioritize around 1000 proteins for more than 4000 ADRs. For a fair comparison, ADRs used in both LAPINE and INPADR were selectively considered for analysis. As a result, we could see that the top 50 predictive proteins predicted by LAPINE showed significantly higher VAPPV and LR (VAPPV = 0.12, LR = 8.20) compared to those predicted INPADR (*t*-test, *P* < 0.001) (Fig. 6C and D). Interestingly, the performance of the prediction model for calculating ADR–protein relation scores affected VAPPV and LR of the top 50 predictive proteins of the corresponding ADR. Specifically, VAPPV and LR of ADRs with high predictive performance (AUROC > 0.7) were significantly higher than those of ADRs that did not (*t*-test, *P* < 0.001) (Supplementary Fig. S5). The verification with the known ADR–drug–protein relationships thus illustrates that the prediction results of LAPINE are more reliable than those of the previous study.

### 3.7 Literature evidence supports high ranked proteins and their enriched KEGG pathways

To further evaluate potential ADR-related proteins predicted by LAPINE, including those that are not known targets, literature-based investigations were performed on the ten highest-ranked proteins. We selected two ADRs that seriously affect patient health for the case study: bradycardia and epilepsy (Chen *et al.*, 2016a; Ovsyshcher and Barold, 2004). In the investigation, all of the top ten predictive proteins for bradycardia and epilepsy were supported by literature evidence. Among them, five predictive proteins for bradycardia and six predictive proteins for epilepsy were confirmed to have a direct causal relation with each ADR (Table 2, Supplementary Table S3). For example, HTR3A, HTR3B and HTR3E are genes that encode proteins for monoamine serotonin (5-HT) receptor subunits. It has been reported that 5-HT receptor stimulation could potentially cause bradycardia. As another example, the overexpression of the Major Vault Protein (MVP) gene was reported in the brain tissues of patients with refractory partial epilepsy, subsequent focal epilepsies after ganglioglioma, and frontal lobe epilepsy.

**Table 2.** Top 10 predictive proteins for Bradycardia

Rank	Symbol	VAPPV	LR	Evidence
1	LYNX1	N/A	N/A	Kessler *et al.* (2017)
2	**HTR3E**	N/A	N/A	
3	**HTR3A**	0.307	3.65	Jeggo *et al.* (2005)
5	**HTR3B**	0.433	6.39	
4	ADRB1	0.274	3.19	Kelley *et al.*, (2018) and Gao *et al.* (2019)
6	CHRNE	0.385	5.11	
8	CHRNA5	0.318	3.83	Riese *et al.* (2014) and Picciotto and Kenny (2013)
10	**CHRNA3**	0.218	2.55	
7	REN	0.175	2.15	Poirier and Tobe (2014)
9	**CYP2D6**	0.273	3.18	Meloche *et al.* (2020) and Sharp *et al.* (2009)

*Note:* Proteins in bold have direct causal evidence for ADR.

Similarly, the literature evidence for the top ten KEGG pathways enriched by the top 50 predictive proteins revealed that 5 of the pathways for bradycardia and 6 of the pathways for epilepsy had evidence that their perturbation can lead directly to each ADR (Table 3, Supplementary Table S4). For example, infusion of acetylcholine is known to cause sinus bradycardia, and variations in genes such as GNB5 can cause bradycardia by affecting the cholinergic responses. As another example, apoptosis-associated molecular mechanisms control neuronal death, a common pathologic hallmark of mesial temporal lobe epilepsy. Therefore, we could see that the predicted proteins and their enriched KEGG pathways for bradycardia and epilepsy are well-supported by previous knowledge or studies.

**Table 3.** Top 10 KEGG pathways enriched by the top 50 predictive proteins for Bradycardia

Rank	Name	*P*-value	Evidence
1	**Neuroactive ligand–receptor interaction**	1.1e–05	Silvani *et al.* (2016) and Herring *et al.* (2008)
2	**Serotonergic synapse**	1.3e–05	Ramage (2001) and N’Diaye *et al.* (2001)
3	**Cholinergic synapse**	2.3e–05	Meyer and Sommers (1988)
4	Nicotine addiction	2.5e–05	Persico (1992)
5	cAMP signaling pathway	2.8e–05	Milanesi *et al.* (2006)
6	Taste transduction	3.4e–05	Horio (2000)
7	Gastric acid secretion	4.6e–05	Cuomo *et al.* (2006) and Rogers and Hermann (1985)
8	**Calcium signaling pathway**	8.1e–05	Graudins *et al.* (2016)
9	Renin secretion	9.1e–05	Adachi *et al.* (2015)
10	**Adrenergic signaling in cardiomyocytes**	1.2e–04	Lymperopoulos *et al.* (2013)

Note: KEGG pathways in bold have direct causal evidence for ADR.

### 3.8 ADR-related proteins are similar among ADRs with the same MeSH term

**Fig. 7.**
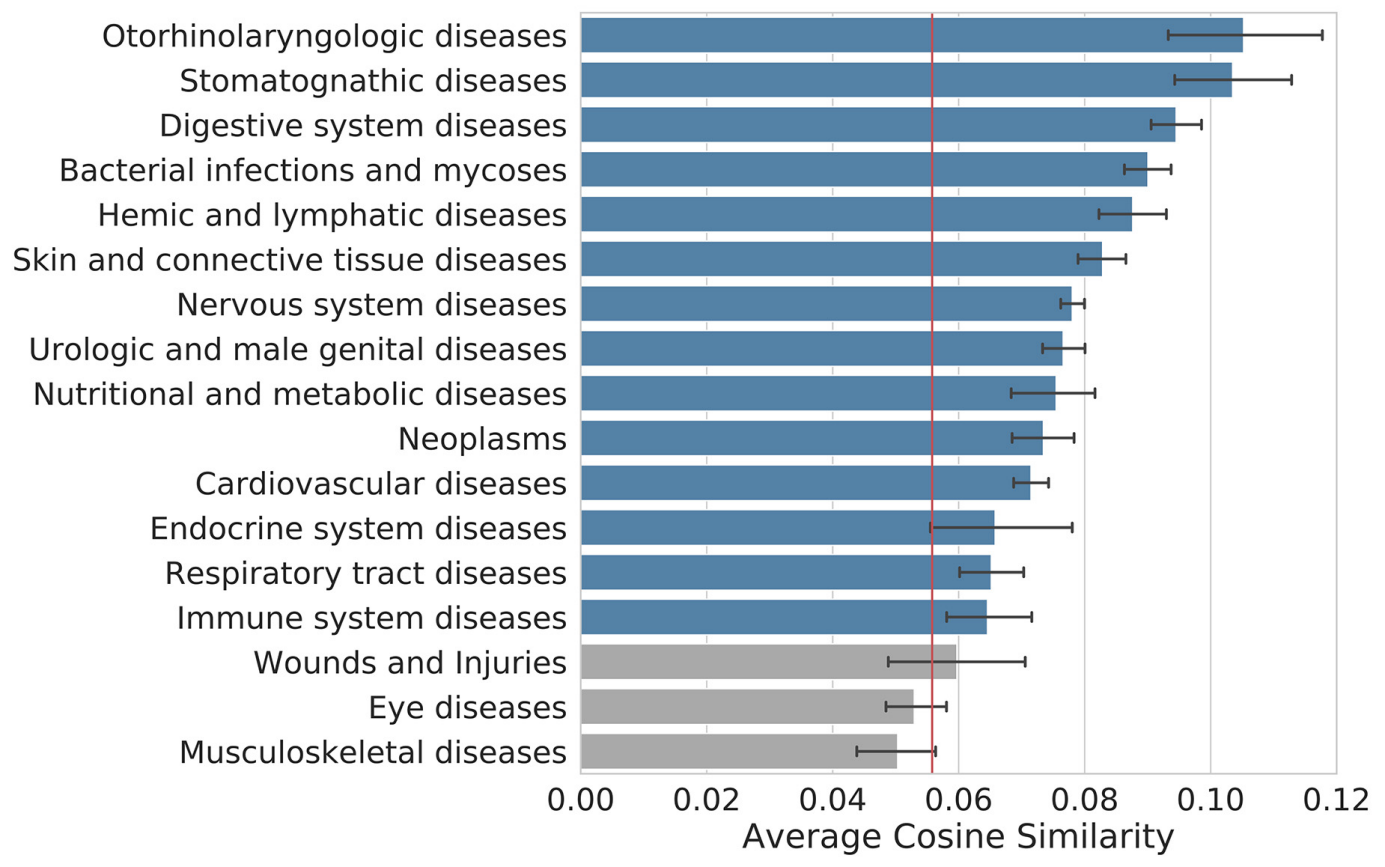
The similarity of related protein score vectors between ADR pairs sharing the same MeSH terms. Bar plots show the cosine similarity of all ADR pairs which share a certain MeSH term. The average cosine similarity of ADR pairs that do not share any mesh terms is indicated by a vertical red line as a baseline. The MeSH terms with a significant difference in the average similarity between the baseline are plotted in the blue bar and others are plotted in the gray bar. The error bar indicates the 95% confidence interval of the average

In order to confirm the reliability and usefulness of predicted scores of ADR-related proteins from another perspective, we investigated the correlation between the physiological similarity and the similarity of protein relation scores of ADR pairs. To obtain physiological similarity, we investigated whether the two ADRs share a certain MeSH term that describes a disease category. For 14 out of 17 MeSH terms for a disease category, ADR pairs that shared MeSH terms showed significantly higher cosine similarity between protein relation scores than ADR pairs that did not share MeSH terms (*t*-test, *P*-value < 0.05) (Fig. 7). The remaining three MeSH terms were not higher than baseline, but not significantly different. Therefore, this result not only showed the reliability of the predicted ADR-related proteins but also showed the possibility that the predicted ADR–protein relation scores can be used as a feature vector representing ADR in other ADR studies.

## 4 Discussion

ADRs are a serious problem in the biomedical sciences, and the identification of target proteins that mediate ADRs may be the key to understanding and preventing ADRs. In this study, we proposed LAPINE to prioritize ADR-related proteins which introduced a network embedding model to the integrated biological network which includes STCs. LAPINE can significantly increase the number of proteins of which relations with ADRs can be predicted. The limited scope of analyzable proteins has been pointed out as one of the important limitations in target-based prediction methods. There was another study that also attempt to extend the scope of considered proteins. But, their approach relies on the small number of known ADR–protein relation data, leading to low predictive reliability. However, LAPINE overcome the limitation of the previous approach by utilizing the drug–ADR relations with the network embedding model and obtained significantly higher reliability compared to the previous approach.

Furthermore, two case studies showed that our predictions are supported by literature evidence. In particular, case studies with the results of KEGG pathway enrichment analysis showed the potential of predicted ADR-related proteins to interpret the mechanism of ADR. Moreover, the similarity of scores between the predicted proteins of pathologically similar ADR pairs suggested the possibility that predicted scores of ADR–protein relations can be utilized in other studies as feature vectors of ADR.

In conclusion, our study provides important information for understanding the mechanism of ADRs and suggests a novel strategy for ADR-related studies based on network embedding. Considering the incompleteness of the available ADR-related data, our achievements are quite meaningful. We expected the increase in quantity and quality of data in the future could sufficiently contribute to improving the accuracy of the prediction. Also, considering the recent powerful performance of deep learning in artificial neural networks, the development of an end-to-end algorithm that integrates the embedding process and predictive model in the field of ADR can be expected.

## Supplementary Material

btac843_Supplementary_Data

## Data Availability

The codes of LAPINE and codes for downloading all public data used in LAPINE are freely available at Github (https://github.com/rupinas/LAPINE) and Figshare (https://figshare.com/articles/software/LAPINE/21750245).
