# Supplementary material for: Large-scale prediction of adverse drug reactions-related proteins with network embedding
Source: Bioinformatics. 2022 Dec 29;39(1):btac843. doi: 10.1093/bioinformatics/btac843 (PMC9825773; doi:10.1093/bioinformatics/btac843)
Supplement: btac843_Supplementary_Data [file btac843_supplementary_data.pdf]

I. Supplementary Data

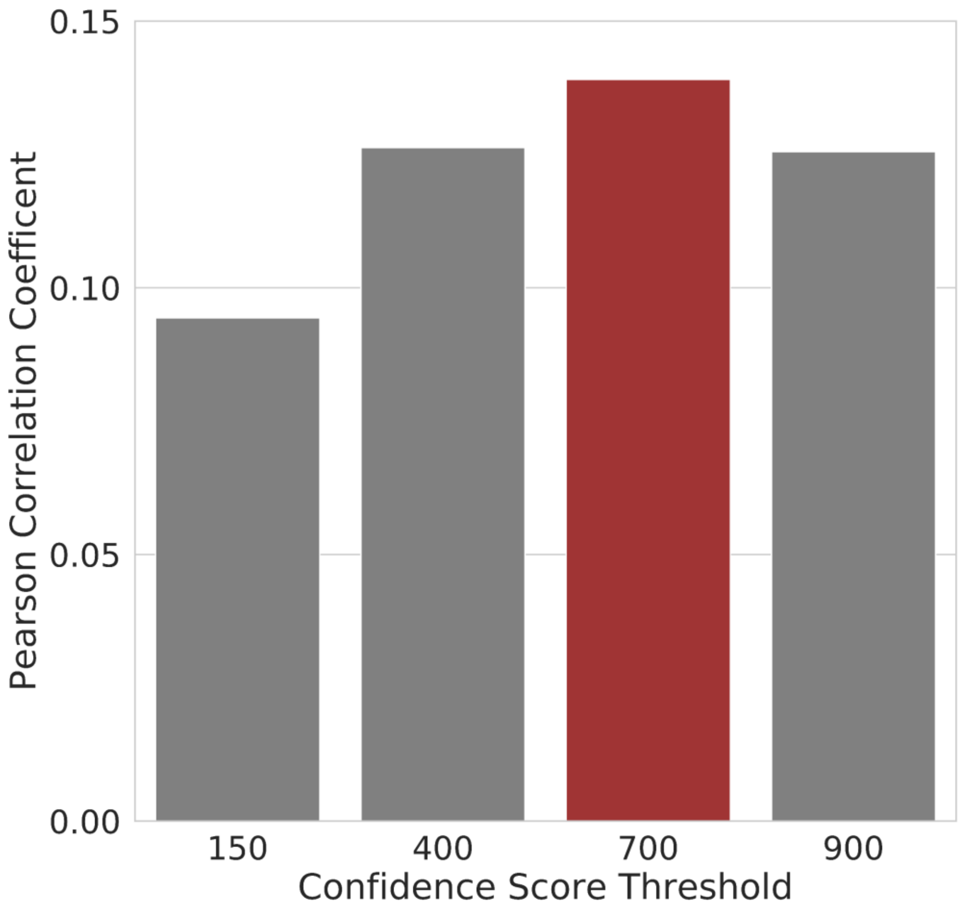

Fig. S1. Pearson correlation coefficient between ADR similarity and target similarity of drug pairs according to threshold of confidence score for protein-protein interactions and drug-protein interactions. Threshold of confidence score is divided into 4 categories suggested by the author of the database; low confidence(150), medium confidence(400), high confidence(700), highest confidence(900). Jaccard similarity between target vector and ADR vector were used for compute ADR similarity and target similarity.

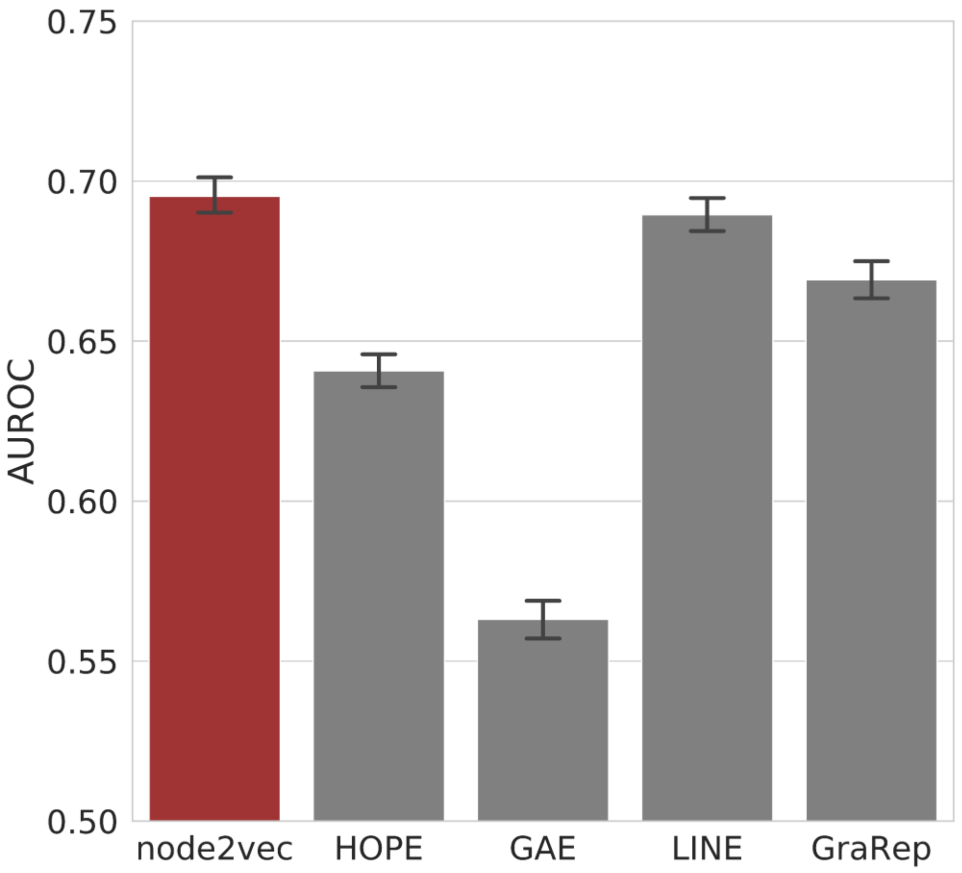

Fig. S2. Effects of graph embedding methods on ADR predictions. Performance of ADR predictions measured with 10-fold cross-validation. The error bar indicates the 95% confidence interval over cross-validation.

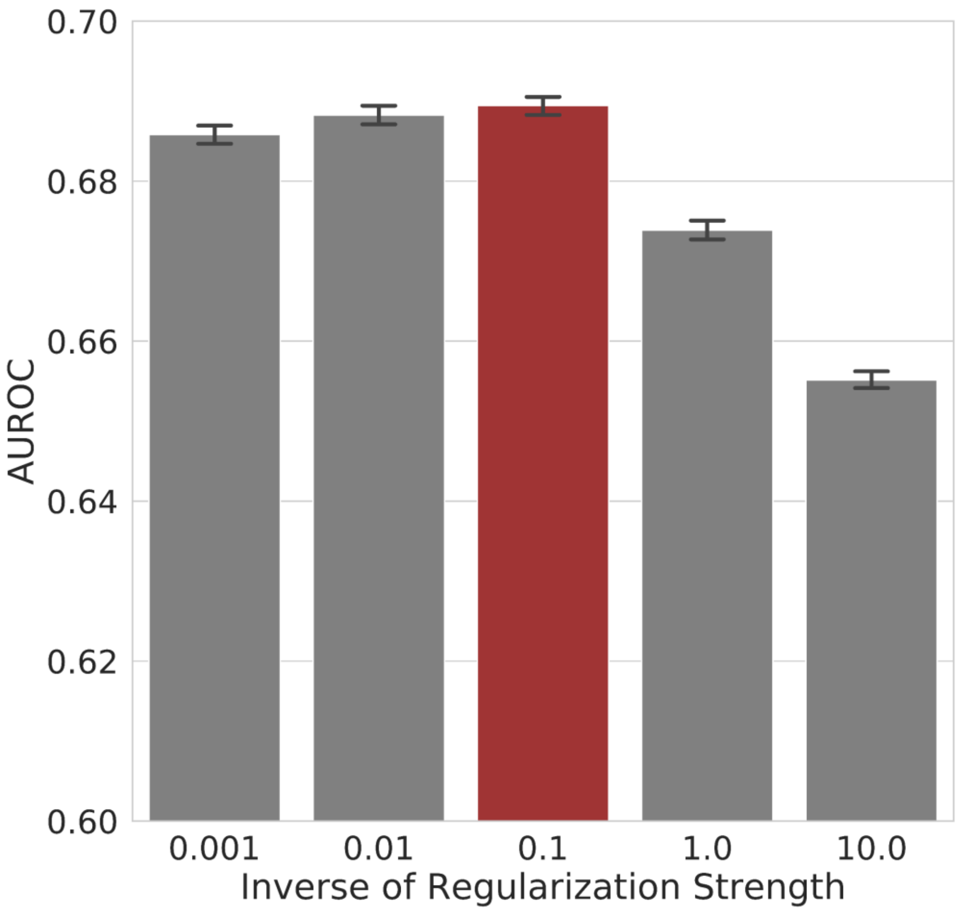

Fig. S3. Effects of regularization strength of logistic regression on ADR predictions. Performance of ADR predictions measured with 10-fold cross-validation. The error bar indicates the 95% confidence interval over cross-validation.

	Drug binds to the protein	Drug not binds to the protein
Drug has the ADR	True positive (TP)	False negative (FN)
Drug has not the ADR	False positive (FP)	True negative (TN)

Fig. S4. Confusion matrix of drugs categorized by the related ADR and binding protein. Using binding to a specific protein as a test for whether a drug has ADR, all drugs can be classified into one of four cases in the confusion matrix (TP, FN, FP, TN).

Table S1. Effects of hyperparameter (p, q) of node2vec on ADR predictions.

**log(p)**	**log(q)**	**AUROC**
**-2**	-2	0.6904±0.0057
**-2**	-1	0.6901±0.0057
**-2**	0	0.6876±0.0059
**-2**	1	0.688±0.0059
**-2**	2	0.6858±0.0059
**-1**	-2	0.6906±0.0058
**-1**	-1	0.6888±0.0058
**-1**	0	0.6888±0.0058
**-1**	1	0.6872±0.0059
**-1**	2	0.6895±0.0058
**0**	-2	0.6907±0.0059
**0**	-1	0.6885±0.0057
**0**	0	0.6914±0.0057
**0**	1	0.6890±0.0058
**0**	2	0.6883±0.0058
**1**	-2	0.6903±0.0058
**1**	-1	0.6947±0.0057
**1**	0	0.6906±0.0058
**1**	1	0.6857±0.0058
**1**	2	0.6838±0.0058
**2**	-2	0.6890±0.0058
**2**	-1	<u>0.6953±0.0057</u>
**2**	0	0.6920±0.0057
**2**	1	0.6913±0.0057
**2**	2	0.6889±0.0057

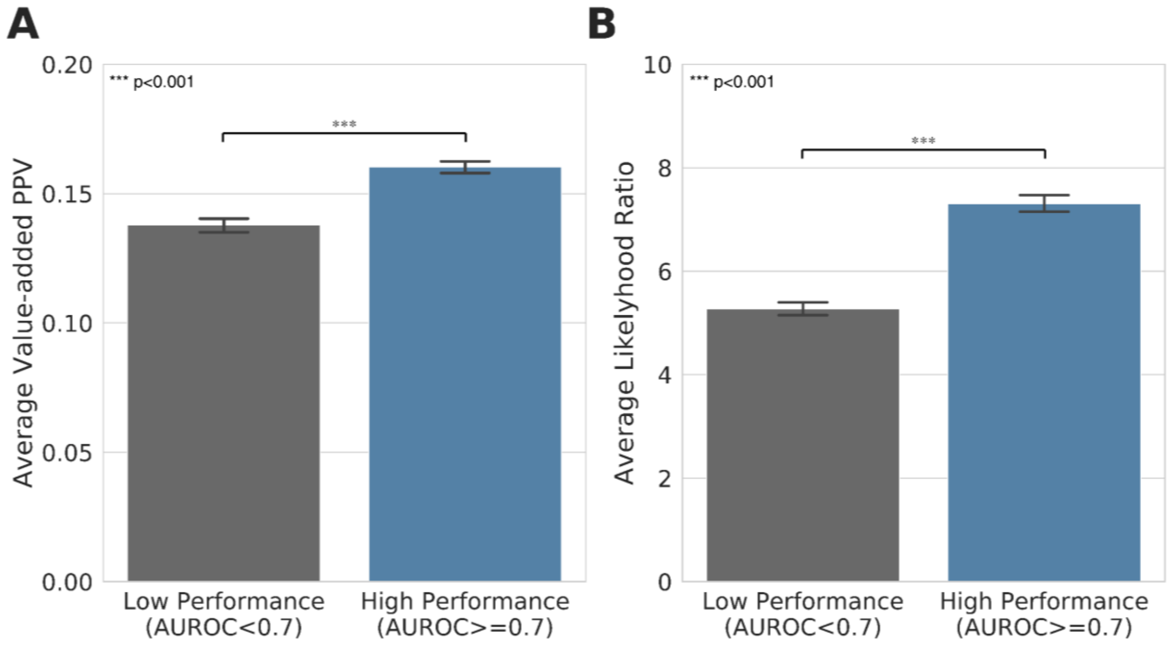

Fig. S5. Effect of ADR prediction model performance on VAPPV and LR of top 50 predictive proteins for the ADR. (A, B) Bar plots showing the average VAPPV and LR of the top 50 predicted proteins for two ADR groups according to the performance of their prediction models. The error bar indicates the 95% confidence interval of the average.

Table S2. Common pharmacological property of the top 10 drug pairs with the highest similarity in low-dimensional representation with low ADR similarity (0.0~0.2).

Drug A	Drug B	Representation similarity	ADR similarity	Common pharmacological property
Epinephrine	Norepinephrine	0.936	0.115	catecholamine family
Flurazepam	Quazepam	0.930	0.155	benzodiazepines
Quazepam	Temazepam	0.929	0.070	benzodiazepines
Bromazepam	Quazepam	0.929	0.107	benzodiazepines
Bromazepam	Estazolam	0.927	0.186	benzodiazepines
Flumazenil	Quazepam	0.918	0.060	benzodiazepines
thiothixene	Iloperidone	0.917	0.140	antipsychotic drugs
Chloramphenicol	Papaverine	0.914	0.095	-
Pramipexole	Iloperidone	0.913	0.182	dopamine agonist, antagonist
Dorzolamide	Hydroflumethiazide	0.909	0.076	hypertension teatment

Table S3. Top 10 predictive proteins for epilepsy

Rank	Symbol	VAPPV	LR	Reference
1	**HTR3A**	0.330	3.979	Zhao *et al.*, 2018
5	**HTR3E**	N/A	N/A	
2	KATNAL2	0.124	1.658	Kurul *et al.*, 2021
3	CD27	0.374	4.969	Rubio *et al.*, 2016
4	CD70	N/A	N/A	
6	**SLC7A11**	0.290	3.316	Robert *et al.*, 2015; Sørensen *et al.*, 2018
7	SLC6A4	0.358	4.591	Cárdenas-Rodríguez *et al.*, 2020
8	ABCC1	0.327	3.937	Van Vliet *et al.*, 2005; Dombrowski *et al.*, 2001
9	**MVP**	N/A	N/A	Sisodiya *et al.*, 2003; Liu *et al.*, 2011; Aronica *et al.*, 2003
10	**CASP3**	0.190	2.162	Henshall *et al.*, 2005

*Proteins in bold have direct causal evidence for ADR

Table S4. Top 10 KEGG pathways enriched by the top 50 predictive proteins for epilepsy

Rank	Name	P-value	Reference
1	**Serotonergic synapse**	3.6e-10	Richerson and Buchanan, 2011
2	**Apoptosis**	6.4e-08	Henshall and Engel, 2013
3	**Taste transduction**	5.5e-05	Heckmann *et al.*, 2003
4	Drug metabolism	1.5e-04	Hedges *et al.*, 2003
5	Legionellosis	3.5e-04	Johnson *et al.*, 1984
6	Pentose and glucuronate interconversions	3.3e-03	-
7	MicroRNAs in cancer	6.6e-03	You *et al.*, 2012
8	**Cocaine addiction**	6.7e-03	Kopppel *et al.*, 1996
9	**Alcoholism**	1.0e-02	Hillborm *et al.*, 2003
10	**Amphetamine addiction**	q.3e-02	Alldredege *et al.*, 1989

*KEGG pathways in bold have direct causal evidence for ADR

Table S5. The summary of previous ADR-protein relation prediction studies

Name	Method	#Protein	#ADR	Score type	Supervised learning[***]
Khun et al., 2013	Fisher's exact test	296	1,428	q-value	No
Chen et al., 2016	Random walk with restart	1,153	4,192	probability	Yes
Letswaart et al., 2020	Random forest	184	321[*]	Gini score	No
Smit et al., 2021	Fisher's exact test	100	3,278	corrected p-value	No
Galletti et al., 2022	Ensemble model[**]	13,090	84	probability	Yes
LAPINE (our method)	Logistic regression	13,633	4,085	z-score	No

* ADR defined with the high level group term (HLGT)
** SVM, Neural network, Random forest
*** The method used known ADR-protein relations to learn the prediction model

II. References

Alldredge,B.K. *et al.* (1989) Seizures associated with recreational drug abuse. *Neurology*, **39**, 1037–1039.

Aronica,E. *et al.* (2003) Overexpression of the human major vault protein in gangliogliomas. *Epilepsia*, **44**, 1166–1175.

Cárdenas-Rodríguez,N. *et al.* (2020) Genetic variations associated with pharmacoresistant epilepsy (Review). *Mol. Med. Rep.*, **21**, 1685–1701.

Chen,X. et al. (2016) Large-scale identification of adverse drug reaction-related proteins through a random walk model. Sci. Rep., 6, 1–10.

Dombrowski,S.M. *et al.* (2001) Overexpression of multiple drug resistance genes in endothelial cells from patients with refractory epilepsy. *Epilepsia*, **42**, 1501–1506.

Galletti,C. et al. (2022) Prediction of Adverse Drug Reaction Linked to Protein Targets Using Network-Based Information and Machine Learning. Front. Bioinform. 2: 906644. doi: 10.3389/fbinf

Heckmann,J.G. *et al.* (2003) Neurological Aspects of Taste Disorders. *Arch. Neurol.*, **60**, 667–671.

Hedges,D. *et al.* (2003) Antipsychotic medication and seizures: a review. *Drugs Today (Barc).*, **39**, 551–557.

Henshall,D.C. and Engel,T. (2013) Contribution of apoptosis-associated signaling pathways to epileptogenesis: Lessons from Bcl-2 family knockouts. *Front. Cell. Neurosci.*, **0**, 110.

Henshall,D.C. and Simon,R.P. (2005) Epilepsy and apoptosis pathways. *J. Cereb. Blood Flow Metab.*, **25**, 1557–1572.

Hillbom,M. *et al.* (2003) Seizures in alcohol-dependent patients: epidemiology, pathophysiology and management. *CNS Drugs*, **17**, 1013–1030.

Hiz Kurul,S. *et al.* (2021) High diagnostic rate of trio exome sequencing in consanguineous families with neurogenetic diseases. *Brain*.

Ietswaart, Robert, et al. (2020) "Machine learning guided association of adverse drug reactions with in vitro target-based pharmacology." EBioMedicine 57 102837.

Johnson,J.D. *et al.* (1984) Neurologic manifestations of Legionnaires' disease. *Medicine (Baltimore).*, **63**, 303–310.

Koppel,B.S. *et al.* (1996) Relation of cocaine use to seizures and epilepsy. *Epilepsia*, **37**, 875–878.

Kuhn,M. et al. (2013) Systematic identification of proteins that elicit drug side effects. Mol. Syst. Biol., 9, 663.

Liu,B. *et al.* (2011) Up-regulation of major vault protein in the frontal cortex of patients with intractable frontal lobe epilepsy. *J. Neurol. Sci.*, **308**, 88–93.

Richerson,G.B. and Buchanan,G.F. (2011) The serotonin axis: Shared mechanisms in seizures, depression and SUDEP. *Epilepsia*, **52**, 28.

Robert,S.M. *et al.* (2015) SLC7A11 expression is associated with seizures and predicts poor survival in patients with malignant glioma. *Sci. Transl. Med.*, **7**.

Rubio,M. *et al.* (2016) Analysis of endocannabinoid signaling elements and related proteins in lymphocytes of patients with Dravet syndrome. *Pharmacol. Res. Perspect.*, **4**, 220.

Sisodiya,S.M. *et al.* (2003) Major vault protein, a marker of drug resistance, is upregulated in refractory epilepsy. *Epilepsia*, **44**, 1388–1396.

Smit,I.A. et al. (2021) Systematic Analysis of Protein Targets Associated with Adverse Events of Drugs from Clinical Trials and Postmarketing Reports. Chem. Res. Toxicol., 34, 365–384.

Sørensen,M.F. *et al.* (2018) High expression of cystine-glutamate antiporter xCT (SLC7A11) is an independent biomarker for epileptic seizures at diagnosis in glioma. *J. Neurooncol.*, **138**, 49–53.

Van Vliet,E.A. *et al.* (2005) Expression of multidrug transporters MRP1, MRP2, and BCRP shortly after status epilepticus, during the latent period, and in chronic epileptic rats. *Epilepsia*, **46**, 1569–1580.

You,G. *et al.* (2012) Significance of miR-196b in tumor-related epilepsy of patients with gliomas. *PLoS One*, **7**.

Zhao,H. *et al.* (2018) 5-HT3 Receptors: A Potential Therapeutic Target for Epilepsy. *Curr. Neuropharmacol.*, **16**.
